# MRI biomarkers of freezing of gait development in Parkinson’s disease

**DOI:** 10.1038/s41531-022-00426-4

**Published:** 2022-11-15

**Authors:** Elisabetta Sarasso, Silvia Basaia, Camilla Cividini, Tanja Stojkovic, Iva Stankovic, Noemi Piramide, Aleksandra Tomic, Vladana Markovic, Elka Stefanova, Vladimir S. Kostic, Massimo Filippi, Federica Agosta

**Affiliations:** 1 Neuroimaging Research Unit, Division of Neuroscience, IRCCS San Raffaele Scientific Institute, Milan, Italy; 2 Clinic of Neurology, Faculty of Medicine, University of Belgrade, Belgrade, Serbia; 3 Vita-Salute San Raffaele University, Milan, Italy; 4 Neurology Unit, IRCCS San Raffaele Scientific Institute, Milan, Italy; 5 Neurorehablitation Unit, IRCCS San Raffaele Scientific Institute, Milan, Italy; 6 Neurophysiology Service, IRCCS San Raffaele Scientific Institute, Milan, Italy

**Keywords:** Predictive markers, Parkinson's disease

## Abstract

This study investigated longitudinal clinical, structural and functional brain alterations in Parkinson’s disease patients with freezing of gait (PD-FoG) and in those developing (PD-FoG-converters) and not developing FoG (PD-non-converters) over two years. Moreover, this study explored if any clinical and/or MRI metric predicts FoG development. Thirty PD-FoG, 11 PD-FoG-converters and 11 PD-non-converters were followed for two years. Thirty healthy controls were included at baseline. Participants underwent clinical and MRI visits. Cortical thickness, basal ganglia volumes and functional network graph metrics were evaluated at baseline and over time. In PD groups, correlations between baseline MRI and clinical worsening were tested. A ROC curve analysis investigated if baseline clinical and MRI measures, selected using a stepwise model procedure, could differentiate PD-FoG-converters from PD-non-converters. At baseline, PD-FoG patients had widespread cortical/subcortical atrophy, while PD-FoG-converters and non-converters showed atrophy in sensorimotor areas and basal ganglia relative to controls. Over time, PD-non-converters accumulated cortical thinning of left temporal pole and pallidum without significant clinical changes. PD-FoG-converters showed worsening of disease severity, executive functions, and mood together with an accumulation of occipital atrophy, similarly to PD-FoG. At baseline, PD-FoG-converters relative to controls and PD-FoG showed higher global and parietal clustering coefficient and global local efficiency. Over time, PD-FoG-converters showed reduced parietal clustering coefficient and sensorimotor local efficiency, PD-non-converters showed increased sensorimotor path length, while PD-FoG patients showed stable graph metrics. Stepwise prediction model including dyskinesia, postural instability and gait disorders scores and parietal clustering coefficient was the best predictor of FoG conversion. Combining clinical and MRI data, ROC curves provided the highest classification power to predict the conversion (AUC = 0.95, 95%CI: 0.86–1). Structural MRI is a useful tool to monitor PD progression, while functional MRI together with clinical features may be helpful to identify FoG conversion early.

## Introduction

Freezing of gait (FoG) is a disabling phenomenon affecting many Parkinson’s disease (PD) patients with the progression of the disease^1,2^. Despite the great research effort, the pathophysiology underlying FoG is still debated. Current evidence supports the multifactorial nature of FoG, suggesting that the alteration of motor, cognitive and behavioral resources might contribute to FoG manifestation as a consequence of fronto-striatal failure^3–7^.

Several MRI studies reported the presence of widespread brain cortical and subcortical grey matter (GM) damage and functional alterations in PD-FoG patients^3,4,8–14^. Consistent findings have widely demonstrated fronto-parietal atrophy in FoG patients relative to those without and healthy controls resulting in executive-attentive and visuospatial dysfunctions, and temporo-parietal cortical alterations being responsible for altered self-body sensorimotor perception and motor planning^8,15,16^. Moreover, evidence reported that reduced volume of basal ganglia in PD-FoG patients is responsible for impaired gait automaticity and motor control^4,5,8^. Many studies have reported reduced resting-state functional connectivity (FC) in default-mode, executive-attentive, visual and sensorimotor networks in PD-FoG patients relative to patients without FoG and healthy controls^17–20^. Both increased and decreased FC was found between cortico-subcortical and cerebellar structures suggesting that FC changes might be related both to neurodegeneration and compensatory mechanisms^8,21–23^. Graph analysis is a relatively recent tool useful to characterize the functional brain network topology in terms of global and local network organization, which has been found to be disrupted in PD^24,25^. Few recent studies using graph analysis^24,26^ suggested an altered FC network organization of frontal areas and of the dorsal attention network in PD-FoG patients relative to PD without FoG and healthy controls.

To date, several studies suggested the presence of clinical motor and non-motor predictors of FoG development, including for instance gait and balance deficits, cognitive changes, mood alterations, hyposmia and REM sleep behavior disorders^27–30^. On the other hand, very few studies provided longitudinal MRI data to explore the progression of PD-FoG patients and even fewer MRI studies monitored over time PD patients developing FoG in order to identify predictive brain signs of FoG onset^31,32^. Preliminary findings showed that baseline white matter hyperintensities and midbrain dysfunction might predispose for higher risk of developing FoG over time^31,32^. Moreover, one study has explored the progression of both structural and functional brain alterations in PD-FoG focusing on the thalamus and suggested that a model including thalamic local volume inflation measures predicts FoG conversions with good accuracy^33^. Within this framework, the aims of this study were: 1) to investigate whole-brain cortical and subcortical GM changes and functional network properties at baseline and over 1- and 2-year follow-up in PD-FoG patients, in patients developing FoG (PD-FoG-converters) and in PD-non-converters; and 2) to identify the best prediction model of FoG development combining both clinical and MRI features.

## Results

### Clinical results

At baseline, patients and healthy controls were similar for age, sex, education, and handedness. The three groups of patients were comparable for all the above-mentioned variables and also for age at onset, disease duration, HY, UPDRS-III, NMS-Q, LEDD and for all cognitive measures at baseline (Table 1). Relative to healthy controls, PD-FoG patients performed worse in all cognitive domains, PD-FoG-converters in attention and language functions, and PD-non converters in language and global cognition (Table 1). Relative to PD-non-converters, PD-FoG patients showed higher values of the postural instability and gait disorders (PIGD) subscore at UPDRS, more severe lower-limb bradykinesia and lower-limb/axial rigidity according to UPDRS-III subscores, more dyskinesia and fluctuations according to UPDRS-IV subscores and higher values at the RBDSQ. Relative to PD-FoG-converters, PD-FoG patients showed higher PIGD and RBDSQ scores. Relative to PD-non-converters, PD-FoG-converters showed greater PIGD, more severe limb/axial rigidity and more frequent dyskinesia.

**Table 1:** Demographic and clinical characteristics at study entry in healthy controls (HC), PD-non converters, PD-FoG-converters and PD-FoG patients and comparisons between groups.

Variables	HC	PD-non converters	PD-FoG-converters	PD-FoG	*P* overall	p: PD-non converters vs HC	p: PD-FoG-converters vs HC	p: PD-FoG vs HC	p: PD-non converters vs PD-FoG-converters	p: PD-non converters vs PD-FoG	p: PD-FoG-converters vs PD-FoG
*N*	30	11	11	30							
Age at MRI [years]	63.67 ± 8.85 (46.00–78.00)	63.84 ± 8.46 (53.00–83.00)	61.86 ± 7.13 (47.00–72.00)	63.81 ± 6.78 (49.00–81.00)	0.89	1.00	1.00	1.00	1.00	1.00	1.00
Sex [Men/Women]	21/9	6/5	7/4	22/8	0.70	0.46	0.72	1.00	1.00	0.28	0.70
Education [years]	12.87 ± 2.85 (8.00-16.00)	12.09 ± 2.77 (8.00–16.00)	11.64 ± 2.34 (8.00–16.00)	11.90 ± 2.72 (4.00–16–00)	0.43	1.00	0.82	1.00	1.00	1.00	1.00
Handedness [Right/Left/ Both]	26/4/0	9/2/0	10/1/0	29/0/1	0.48	0.65	1.00	0.08	1.00	0.05	0.21
Age at onset [years]	-	56.18 ± 7.03 (43.00–71.00)	51.00 ± 7.35 (42.00–63.00)	53.90 ± 7.75 (41.00–71.00)	0.31	-	-	-	0.37	1.00	1.00
PD duration [years]	-	7.66 ± 3.02 (4.00-14.00)	10.86 ± 4.63 (4.00-17.00)	9.91 ± 4.37 (3.00-22.00)	0.17	-	-	-	0.24	0.35	1.00
FoG-Q (max = 24)	-	-	-	9.40 ± 3.01 (5.00–18.00)	-	-	-	-	-	-	-
H&Y (max = 5)	-	1.91 ± 0.86 (1.00-3.00)	2.54 ± 0.65 (1.00-3.00)	2.63 ± 0.54 (1.00-3.00)	0.12	-	-	-	0.38	0.03	0.78
UPDRS-II (max = 52)	-	9.73 ± 6.61 (2.00–19.00)	12.73 ± 3.72 (7.00–19.00)	14.67 ± 4.67 (3.00–23.00)	0.01	-	-	-	0.15	0.006	0.08
UPDRS-III (max = 132)	-	32.82 ± 17.20 (13.00–62.00)	43.64 ± 8.10 (29.00–58.00)	47.10 ± 9.55 (27.00–76.00)	0.05	-	-	-	0.61	0.05	1.00
UPDRS PIGD score (max = 20)	-	3.00 ± 0.77 (2.00–4.00)	5.18 ± 2.04 (3.00–10.00)	7.33 ± 2.02 (4.00-12.00)	<0.001	-	-	-	0.002	<0.001	0.004
UPDRS-III LL bradykinesia (max = 16)	-	6.09 ± 3.60 (2.00–10.00)	8.36 ± 2.16 (4.00–10.00)	9.00 ± 1.97 (3.00–13.00)	0.07	-	-	-	0.13	0.03	0.48
UPDRS-III LL/axial Rigidity (max = 12)	-	4.27 ± 2.28 (2.00–8.00)	5.91 ± 1.30 (3.00–8.00)	6.00 ± 1.58 (2.00–8.00)	0.06	-	-	-	0.04	0.03	0.95
UPDRS-IV Dyskinesia (max = 8)	-	0.27 ± 0.47 (0.00–1.00)	1.64 ± 1.75 (0.00–6.00)	1.40 ± 1.13 (0.00–4.00)	0.009	-	-	-	0.01	0.003	0.96
UPDRS-IV Fluctuations (max = 12)	-	0.82 ± 1.47 (0.00–4.00)	1.55 ± 1.04 (0.00–3.00)	1.93 ± 1.05 (0.00–4.00)	0.04	-	-	-	0.13	0.02	0.30
RBDSQ (max = 13)	-	2.55 ± 1.97 (1.00–6.00)	2.82 ± 1.54 (1.00–5.00)	5.27 ± 3.22 (1.00–12.00)	0.007	-	-	-	0.14	0.008	0.02
NMS-Q (max = 30)	-	4.64 ± 2.64 (1.00–9.00)	4.36 ± 2.80 (0.00–10.00)	5.60 ± 2.81 (0.00–10.00)	0.39	-	-	-	0.84	0.36	0.22
Levodopa equivalent dose [mg]	-	690.46 ± 390.02 (150.00–1450.00)	893.64 ± 345.67 (250.00–1530.00)	955.83 ± 335.21 (280.00–1930.00)	0.08	-	-	-	0.44	0.07	1.00
*Global cognition*											
ACE-R total (max = 100)	95.67 ± 3.28 (87.00-100.00)	87.09 ± 8.53 (70.00-98.00)	88.91 ± 9.71 (65.00-99.00)	85.87 ± 6.92 (71.00-97.00)	<0.001	0.01	0.10	<0.001	1.00	1.00	0.68
*Memory*											
RAVLT, immediated recall (max = 75)	43.73 ± 9.33 (28.00–64.00)	36.82 ± 11.81 (24.00–58.00)	39.72 ± 8.08 (28.00–50.00)	36.07 ± 10.88 (17.00–55.00)	0.04	0.34	1.00	0.04	1.00	1.00	1.00
RAVLT, delayed recall (max = 15)	8.53 ± 2.54 (4.00–13.00)	6.09 ± 2.80 (2.00–10.00)	7.55 ± 2.66 (3.00–11.00)	5.57 ± 2.94 (0.00–12.00)	0.001	0.12	1.00	0.001	1.00	1.00	0.36
PRM [% correct]	78.19 ± 12.17 (35.50–91.67)	67.82 ± 13.40 (50.00–92.00)	76.54 ± 27.97 (0.00–100.00)	68.76 ± 18.37 (0.00–95.00)	0.01	0.12	1.00	0.06	0.17	1.00	0.17
SRM [% correct]	73.50 ± 9.30 (55.00–90.00)	68.18 ± 13.47 (50.00–85.00)	70.46 ± 16.65 (45.00–85.00)	65.17 ± 6.21 (0.00–90.00)	0.18	1.00	1.00	0.25	1.00	1.00	0.99
*Language*											
BNT Total (max = 60)	57.83 ± 1.88 (53.00–60.00)	56.00 ± 4.10 (46.00–60.00)	57.70 ± 1.16 (56.00–59.00)	53.97 ± 9.97 (5.00–60.00)	0.03	1.00	1.00	0.02	1.00	1.00	0.54
ACE-R, language (max = 26)	25.90 ± 0.31 (25.00–26.00)	23.55 ± 2.98 (17.00–26.00)	23.64 ± 1.86 (21.00–26.00)	22.83 ± 2.77 (14.00–26.00)	<0.001	0.01	0.003	<0.001	1.00	1.00	1.00
*Fluency*											
Semantic fluency (max = N.A.)	19.57 ± 4.45 (12.00–30.00)	15.64 ± 5.60 (8.00–23.00)	19.45 ± 5.12 (11.00–28.00)	15.00 ± 3.25 (10.00–23.00)	<0.001	0.11	1.00	<0.001	0.44	1.00	0.06
Phonemic fluency (max = N.A.)	37.47 ± 9.05 (20.00–59.00)	33.00 ± 12.39 (15.00–53.00)	34.18 ± 13.60 (17.00–58.00)	30.50 ± 8.48 (11.00–47.00)	0.08	1.00	1.00	0.06	1.00	1.00	1.00
*Executive functions*											
Digit backward (max = 11)	6.43 ± 1.81 (4.00–11.00)	5.80 ± 1.81 (4.00–10.00)	5.82 ± 3.03 (3.00–11.00)	5.07 ± 1.72 (0.00–11.00)	0.02	1.00	0.16	0.03	1.00	1.00	1.00
Stroop, interference [total correct] (max = 60)	38.47 ± 11.36 (18.00–60.00)	34.00 ± 13.00 (14.00–51.00)	28.91 ± 6.80 (20.00–41.00)	23.53 ± 9.35 (7.00–50.00)	<0.001	1.00	0.27	<0.001	1.00	0.10	0.92
IED [total errors] (max = 450)	38.03 ± 23.01 (7.00–91.00)	51.18 ± 21.08 (11.00–72.00)	64.00-49.91 (0.00–200.00)	63.57 ± 52.52 (8.00–225.00)	0.054	0.36	0.25	0.15	1.00	1.00	1.00
*Attention*											
Digit ordering [max. span] (max = 12)	6.97 ± 2.17 (4.00–12.00)	5.36 ± 2.16 (2.00–9.00)	4.91 ± 1.64 (2.00–8.00)	4.60 ± 1.65 (2.00–8.00)	<0.001	0.26	0.07	<0.001	1.00	1.00	1.00
Letter cancellation correct (max = 40)	26.73 ± 4.95 (20.00–40.00)	21.80 ± 4.89 (15.00–30.00)	20.20 ± 2.39 (15.00–23.00)	20.10 ± 6.13 (0.00–34.00)	<0.001	0.048	0.002	<0.001	1.00	1.00	1.00
*Visuospatial abilities*											
Hooper (max = 30)	22.60 ± 3.42 (13.00–30.00)	18.14 ± 6.31 (10.00–27.00)	16.14 ± 6.32 (2.00–23.00)	17.67 ± 6.21 (5.00–29.00)	0.002	0.19	0.01	0.01	1.00	1.00	1.00
ACE-R, visuospatial (max = 16)	15.73 ± 0.45 (15.00–16.00)	15.27 ± 1.10 (13.00–16.00)	14.91 ± 1.64 (12.00–16.00)	14.70 ± 1.42 (12.00–16.00)	0.03	1.00	1.00	0.02	1.00	1.00	1.00
*Mood/behavior*											
HAMA (max = 56)	3.20 ± 3.20 (0.00–11.00)	5.91 ± 4.64 (0.00–16.00)	4.00 ± 4.15 (0.00–11.00)	7.57 ± 6.00 (0.00–21.00)	0.02	0.71	1.00	0.02	1.00	1.00	0.48
HDRS (max = 50)	2.77 ± 4.27 (0.00–15.00)	5.00 ± 5.33 (0.00–15.00)	4.64 ± 3.29 (0.00–10.00)	6.47 ± 5.81 (0.00–20.00)	0.01	0.28	0.39	0.01	1.00	1.00	1.00
Apathy Scale (max = 72)	1.73 ± 3.17 (0.00–11.00)	13.09 ± 8.62 (0.00–28.00)	8.91 ± 7.23 (0.00–21.00)	11.70–9.16 (0.00–28.00)	<0.001	<0.001	0.02	<0.001	1.00	1.00	1.00

Values are reported as mean ± standard deviation (range) or absolute for continuous and categorical variables, respectively. Differences between PD patients and healthy controls and between PD groups at baseline were assessed using the Kruskal-Wallis test; *P* values were adjusted for multiple group comparisons at level 0.05. Two-sided *p* value <0.05 was considered for statistical significance. *ACE-R* Addenbrooke’s Cognitive Examination*-*Revised*, BNT* Boston Naming Test, *FoG-Q* Freezing of gait questionnaire*, HAMA* Hamilton Anxiety Rating Scale*, HC* Healthy controls*, HDRS* Hamilton Depression Rating Scale*, IED* Intra and Extra-Dimensional shifting*, LL* Lower limb*, N.A*. Not available, *NMS-Q* Non-Motor Symptoms Questionnaire, *PIGD* Postural instability and gait disorders*, PD* Parkinson’s disease*,* *PRM* Pattern Recognition Memory*, RAVLT* Rey Auditory Verbal Learning Test*, RBDSQ* REM Sleep Behavior Disorder Screening Questionnaire*, SRM* Spatial Recognition Memory*, UPDRS* Unified Parkinson’s Disease Rating Scale.

Longitudinal clinical results showed a worsening of motor symptoms (UPDRS-III beta=9.64), language functions (semantic fluency beta = −4.15), and mood (HDRS beta=5.72; HAMA beta=4.27) in PD-FoG-converters, and a worsening of motor symptoms (UPDRS-III beta=6.11), global cognition (ACE tot beta=5.18), executive-attentive functions (digit backward beta = −0.99; letter cancellation correct beta = −3.56; phonemic fluency beta = −6.26), memory (RAVLT immediate recall beta = −3.79) and depressive symptoms (HDRS beta=3.90) in PD-FoG. Moreover, the latter group showed significantly higher LEDD over time (beta = 167.28). No significant differences were found in PD-non-converters over time and in group-by-time interaction between groups.

### Baseline structural and functional MRI findings

**Fig. 1:**
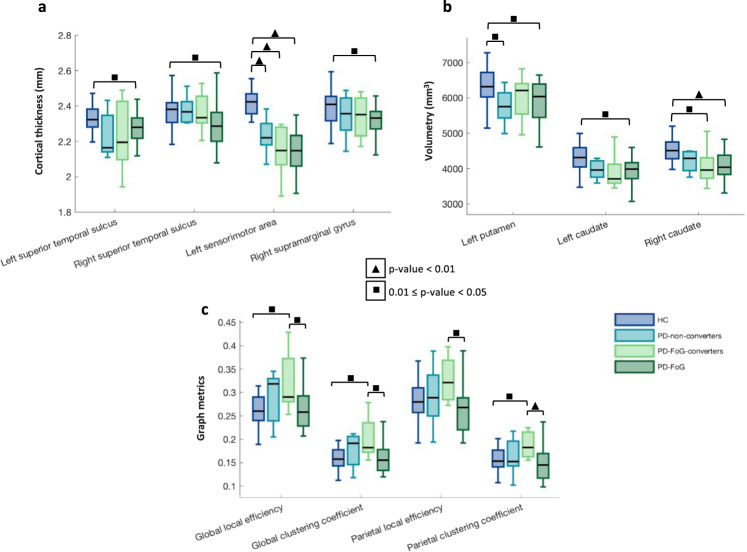
Cortical thickness, basal ganglia volume and functional graph metric differences in PD groups and healthy controls at baseline. **a** Cortical thickness pattern at baseline in PD-FoG, PD-FoG-converters and PD-non-converters patients relative to healthy controls and between each other. **b** Basal ganglia volume at baseline in PD-FoG, PD-FoG-converters and PD-non-converters patients relative to healthy controls and between each other. **c** Graph metric differences at baseline in PD-FoG, PD-FoG-converters and PD-non-converters patients relative to healthy controls and between each other. *P* value < 0.05 was considered for statistical significance corrected for multiple group comparisons. The horizontal lines in each box plot represent, from the bottom to the top, the 25th percentile, the median and 75th percentile; whiskers represent the minimum and maximum values.

PD-FoG patients showed reduced mean thickness of bilateral superior temporal sulci, left sensorimotor (including precentral, postcentral and paracentral areas) and right supramarginal areas (Fig. 1a) and reduced volume of bilateral caudate and left putamen relative to healthy controls (Fig. 1b). Both PD-FoG-converters and PD-non-converters showed reduced mean thickness of left sensorimotor areas relative to healthy controls (Fig. 1a). In addition, PD-FoG-converters showed reduced volume of right caudate relative to healthy controls, while the volume of left putamen was smaller in PD-non-converters relative to healthy controls at baseline (Fig. 1b). fMRI analysis showed that PD-FoG-converters had higher global local efficiency, global clustering and parietal clustering coefficient relative to both PD-FoG cases and healthy controls, and higher parietal local efficiency relative to PD-FoG patients at baseline (Fig. 1c).

### Longitudinal structural and functional MRI findings

**Fig. 2:**
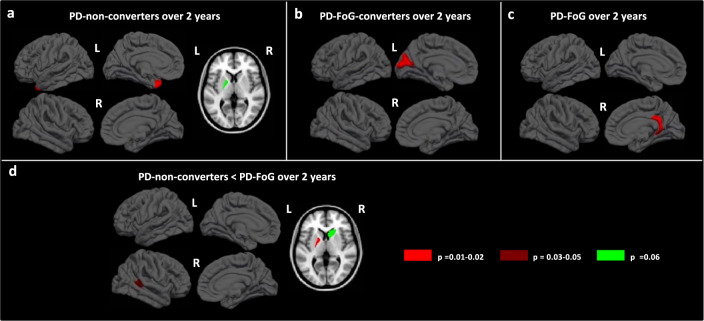
Cortical thinning and basal ganglia atrophy over time in PD groups. **a** Cortical thinning pattern and basal ganglia atrophy over time in PD-non-converters groups; **b** cortical thinning pattern over time in PD-FoG-converters; **c** cortical thinning pattern over time in PD-FoG; and **d** cortical thinning and basal ganglia atrophy over time in PD-FoG relative to PD-non-converters. *P* value < 0.05 was considered for statistical significance corrected for multiple group comparisons. Color bars represent *p* values. Left is left, right is right.

**Fig. 3:**
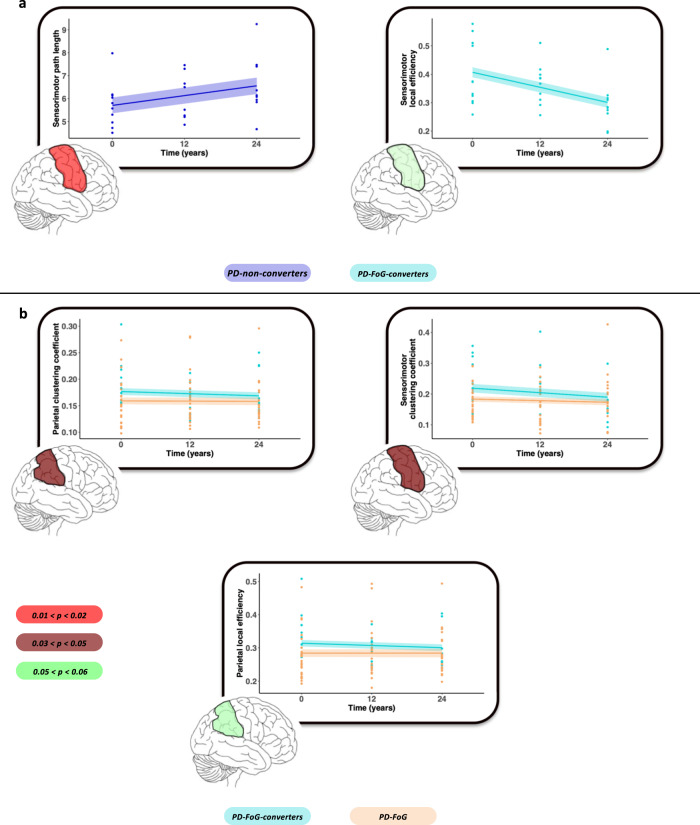
Functional graph metrics changes in PD groups over time. **a** Functional network graph metric changes over time in PD-non-converters; **b** Graph metric changes over time in PD-FoG-converters relative to PD-FoG. *P* value < 0.05 was considered for statistical significance corrected for multiple group comparisons.

Over time, PD-non-converters showed a significant mean cortical thinning of left temporal pole and a trend toward a significant reduced volume of the left pallidum (Fig. 2a). PD-FoG-converters showed a significant mean cortical thinning of left cuneus (Fig. 2b), while PD-FoG patients of the right isthmus cingulate cortex (Fig. 2c). Between-group comparison showed a smaller volume of left pallidum in PD-non-converters relative to PD-FoG over time, and a trend toward a mean cortical thinning of right superior temporal pole and right caudate atrophy (Fig. 2d). In addition, PD-non-converters showed an increased sensorimotor path length (Fig. 3a) and PD-FoG-converters presented a trend toward a significant decrease of sensorimotor local efficiency (Fig. 3a). PD-FoG-converters showed a reduced parietal and sensorimotor clustering coefficient and a trend toward a significant reduction parietal local efficiency relative to PD-FoG patients during follow-up (Fig. 3b).

### Correlations between MRI results and clinical worsening over time

**Fig. 4:**
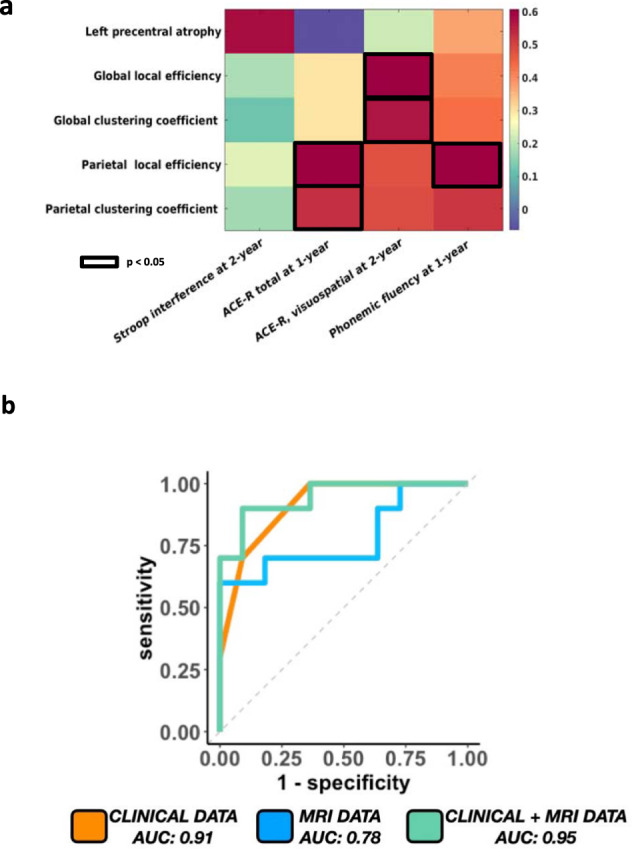
Correlation findings and ROC curve analysis differentiating PD-FoG-converters from PD-non-converters. **a** Spearman’s correlations between baseline MRI metrics (which were found to be significantly different between groups) and worsening of clinical data over 1- and 2-year follow-up in PD-FoG and PD-FoG-converters. *P* value<0.05 was considered for statistical significance corrected for multiple group comparisons. **b** The ROC curve showing the value of clinical (dyskinesia and PIGD scores), MRI (parietal clustering coefficient) and clinical+MRI data in distinguishing PD-FoG-converters and PD-non-converters at baseline.

Correlation analysis showed that in PD-FoG patients cortical atrophy of left precentral gyrus at baseline correlated with worsening of executive functions at 2-year follow-up; lower global local efficiency and global clustering at baseline correlated with worsening of visuo-spatial functions at two years; and lower parietal local efficiency and clustering coefficient at baseline correlated with worsening of global cognition and executive functions at 1-year follow-up (Fig. 4a).

### Stepwise prediction model and ROC curve

The multicollinearity analysis showed that the correlation between all the predictor variables is above −0.9 or below +0.9. Stepwise prediction model, including dyskinesia (*t* = −2.03; *p* = 0.05) and PIGD score (*t* = −2.87; *p* = 0.01), and parietal clustering coefficient (*t* = −2.09; *p* = 0.05) was the best predictor of FoG conversion. Hereafter, ROC curve analysis showed that clinical variables resulting from the stepwise model at baseline distinguished PD-FoG-converters from PD-non-converters with a good accuracy (area under the curve [AUC] = 0.91, 95% confidence interval [CI]: 0.80–1) (Fig. 4b). MRI variables alone had a high power to discriminate PD-FoG-converters from PD-non-converters (AUC = 0.78, 95%CI: 0.56–0.99) (Fig. 4b). Combining clinical and MRI data, ROC curves provided the highest classification power to predict the conversion (AUC = 0.95, 95% CI: 0.86–1) (Fig. 4b). Considering that the PIGD score includes by definition a FoG item, to be sure that the PIGD score was truly predictive without this information, we run again the prediction model after removing the FoG item from the PIGD score and we obtained exactly the same results in the stepwise prediction model and the same values at the ROC curve analysis.

## Discussion

In this study, we investigated the clinical, cortical and subcortical GM and functional brain network alterations over 2-year follow-up in PD-FoG patients, PD-FoG-converters and PD-non-converters. Moreover, we assessed the role of MRI variables together with clinical features in identifying PD-FoG-converters over two years of follow-up. Our study has the main strengths to include PD subgroups matched for confounding variables at baseline and to monitor FoG progression and conversion using both clinical measures and multiparametric MRI metrics longitudinally. Our findings support the important role of structural and functional MRI to monitor disease progression and to detect brain changes that might precede the development of specific clinical features such as FoG.

One recent study^33^ investigated the role of thalamic structural changes in predicting FoG development. Both the global volume and sub-regional shape variations of subcortical structures were investigated suggesting that specific local shape inflation of the thalamus predicted conversion with a good accuracy (AUC = 0.87). In our study, we only performed a global volume analysis and we confirmed that the global volume of the thalamus is not altered in PD-FoG-converters relative to PD-non-converters. At baseline, we observed that PD-FoG patients had a widespread cortical/subcortical atrophy, while both PD-FoG-converters and non-converters showed patterns of GM atrophy only in sensorimotor areas and basal ganglia. These results are in line with the clinical characteristics of the patients. Indeed, despite our PD groups were statistically matched for disease severity and disease duration, PD-FoG patients were in a more advanced stage of the disease presenting a slightly higher UPDRS III score relative to PD-non-converters and cognitive/behavioral alterations relative to healthy controls (Table 1). According to the current literature, it is quite expected to find a widespread cortical thinning in frontal, temporal and parietal areas in moderate to severe patients such as PD-FoG cases having executive-attentive, visuospatial, language and memory deficits^4,15,16,18^, while an initial atrophy of sensorimotor areas and basal ganglia is common in the early/middle phase of the disease^6,11^. Over time, PD-non-converters accumulated atrophy in the temporal pole and in the left pallidum without showing statistically significant clinical changes. A possible explanation could be that this pattern of atrophy might not be sufficient to significantly impact on the clinical status of the patient. Moreover, previous evidence suggested that structural brain alterations could precede the development of clinical changes^11^. On the other hand, PD-FoG-converters showed worsening of disease severity, executive functions, and mood together with an accumulation of occipital atrophy. Considering that PD-FoG-converters and PD-non-converters had comparable neurological (HY, UPDRS-II, UPDRS-III, NMS-Q and RBDSQ) and cognitive characteristics at baseline, the pattern of progression that we observed in PD-FoG-converters might represent a more aggressive disease spreading according to the Braak model^6^. The fast structural changes of posterior cortical areas might be associated with the accumulation of executive-attentive deficits and with the development of FoG, supporting once again the multifactorial nature of this phenomenon^34^. Cortical thickness changes in PD-FoG-converters are similar to the pattern of posterior cortical thinning that we observed in PD-FoG patients (i.e. thinning of the isthmus cingulate). PD-FoG patients also continued to worsen in all cognitive domains and mood. Interestingly, the reduced cortical thickness of the motor cortex in PD-FoG at baseline correlated with the accumulation of executive deficits over 2 years, suggesting the strong interaction between motor and cognitive alterations in PD-FoG patients as previously reported^8^.

Our study investigated also functional topological brain network alterations underlying FoG development. Graph metrics are useful to understand how the brain is topologically organized to support efficient information processes at a local and global level. At baseline, PD-FoG-converters showed higher global and parietal local efficiency and clustering coefficient relative to PD-FoG cases and healthy controls, suggesting an increased functional communication in the parietal network and globally in the brain in PD-FoG-converters. These findings are in line with recent evidence supporting the presence of a cholinergic up-regulation in specific gait control networks (including the parietal lobe) as a compensatory mechanism to counteract aberrant dopaminergic function in PD^35,36^. The pattern of increased parietal clustering coefficient might also represent an attempt to compensate for the incremental structural alteration of posterior cortical areas in PD-FoG-converters^34^. Interestingly the higher parietal clustering coefficient at baseline contributed to differentiate PD-FoG–converters from PD-non-converters (AUC = 0.78), suggesting that a topological functional alteration of the parietal cortex might be considered a red flag preceding FoG development. We also found that specific clinical variables such as a higher PIGD score at the UPDRS and more frequent dyskinesia might contribute to identify PD-FoG-converters relative to PD-non-converters at baseline (AUC = 0.91). The higher PIGD and dyskinesia scores might represent a reduced response to pharmacological therapy that is known to be associated with FoG development. Previous studies already suggested these clinical features as potential predictors of FoG^37,38^. Other studies suggested that also non-motor symptoms including REM sleep disorders, cognitive and mood alterations might contribute to FoG development^27,28,30^, however we did not observe any difference between PD-FoG-converters and PD-non-converters at baseline in our sample. Our study has the main strength to combine clinical and MRI data showing that the highest accuracy of the prediction model (AUC = 0.95) metric could be obtained combining clinical and functional topological MRI data.

Over 2 years, both PD-non-converters and PD-FoG-converters showed a functional topological disruption. The increased sensorimotor path length in the PD-non-converter group and the decreased sensorimotor local efficiency in PD-FoG-converters might represent a reduced ability to transfer information within the sensorimotor network and between the sensorimotor and other networks as a consequence of the disease progression. In addition, PD-FoG-converters likely lost the ability to compensate showing a reduction of parietal clustering coefficient. One might speculate that the progressive loss of this compensatory mechanism at the parietal level could contribute to FoG development. This is in line with recent evidence suggesting that PD-FoG patients showed reduced global and local brain functional efficiency relative to healthy controls and PD without FoG^24^. This hypothesis is supported also by the significant correlations that we found between the lower global local efficiency, global clustering and parietal clustering at baseline and the worsening of attentive/executive functions at 1-year and 2-year follow-up in PD-FoG patients. On the contrary, the increased parietal clustering coefficient at baseline could be also considered a maladaptive process as a consequence of neuronal loss. In any case, the central role of parietal structural and functional alterations in identifying most severe PD cases and PD-FoG is well-established^34,39,40^. This area is crucial for the sensory integration of spatio-temporal inputs, for working memory processes and for movement programming^4,15,16,18,41–43^. Thus, it is easy to understand how the structural/functional disruption of the parietal lobe could contribute to FoG manifestation and to executive-attentive dysfunctions^4,15,16,18,44^. Our findings suggest the importance of studying the parietal functional topological characteristics from the initial stages of the disease in order to detect early functional reorganization that might help foresee FoG development.

This study is not without limitations. First, the samples of PD-FoG-converters and PD-non-converters were quite small and this could have contributed to the absence of group-by-time interaction in the clinical and MRI findings. Moreover, the three groups of PD patients were statistically matched for UPDRS-III although bordering statistical significance (*p* = 0.05). Second, we did not have longitudinal MRI data in healthy subjects that could be useful to control for the aging effects. Third, we used a 1.5 T MRI scanner, which is characterized by a lower spatial resolution and BOLD signal to noise ratio resolution compared with higher field strength scanners. Fourth, PD patients were evaluated in ON status. However, our analysis accounted for both LEDD at baseline and LEDD change over time in order to mitigate the effect of this potential confounding factor. Fourth, a longer follow-up might improve the possibility to monitor FoG conversion and progression; however, to the best of our knowledge, this is the first longitudinal study exploring brain correlates underlying FoG development in PD patients. Fifth, given the small sample size of the PD-FoG-converter and PD-non-converter groups, the prediction model might suffer from overfitting of data. Future studies should verify our preliminary findings in an independent sample.

In conclusion, this study offers novel insights into the mechanisms underlying FoG. We demonstrated that structural MRI is a useful tool to monitor PD progression: PD-non-converters accumulated cortical thinning in the left temporal pole and pallidum without relevant clinical and cognitive changes over 2 years, while PD-FoG-converters showed an accumulation of occipital atrophy similarly to PD-FoG and a worsening of both motor and cognitive/behavioral features. Functional MRI could be considered a useful tool to identify FoG conversion in PD: the increased parietal clustering coefficient at baseline in PD-FoG-converters could represent a marker of FoG development over few years, together with the PIGD score and the presence of dyskinesia. Future large-cohort studies are now warranted to confirm these findings and provide robust early biomarkers of FoG, which can be useful to guide careful drug choice, neurosurgery or tailored physiotherapy approaches targeted to prevention and early management of FoG evoking conditions.

## Methods

### Participants

A sample of 52 idiopathic PD patients^45^ were selected among a larger cohort of 154 PD subjects prospectively recruited at the Clinic of Neurology, School of Medicine, University of Belgrade, Belgrade, Serbia within the framework of an ongoing longitudinal project^11,20,39,46^. After removing nine subjects presenting MRI movement artefacts at one of the time-points, the selected sample included 30 PD-FoG patients, 11 PD-FoG-converters and 11 PD-non-converters, matched for age, sex, education, disease duration and disease severity using the Unified Parkinson’s Disease Rating Scale part III (UPDRS-III, although a trend toward a higher score in PD-FoG relative to PD-non-converters was found, *p* = 0.05). PD-FoG patients had FoG at baseline (according to the criteria reported in the next paragraph), while PD-FoG-converters developed FoG over the 2-year follow-up. Patients received a comprehensive evaluation in ON medication state including neurological, cognitive/behavioral and MRI assessments at study entry and at 1-year and 2-year follow-up. MRI scans were acquired to evaluate regional cortical thickness, basal ganglia volumes and functional network graph metrics alterations. Patients were excluded if they had: Hoehn and Yahr (HY) score > 4 and/or dementia^47^ because they are usually less cooperative and may have some difficulties to stay still into the MRI scanner and to participate to all the study visits; moderate/severe head tremor at rest; cerebrovascular disorders (including vascular parkinsonism) or intracranial masses on routine MRI; history of traumatic brain injury; any other major neurological and medical condition; and MR images with artefacts. Thirty healthy controls matched for age, sex and education, without any neurological, psychiatric, or other disorders, were also recruited among friends and non-consanguineous relatives of patients and by word of mouth and performed clinical cognitive evaluation and MRI assessments at baseline as comparison with PD groups.

Ethical standard committees on human experimentation of IRCCS Ospedale San Raffaele, Milan, Italy and Clinic of Neurology, Faculty of Medicine, University of Belgrade, Serbia approved the study protocol; all participants provided written informed consent prior to study inclusion.

### Neurological evaluations

At study entry and each follow-up visit, an experienced neurologist blinded to MRI results performed clinical assessments. Patients were examined in ON state (i.e., period when the dopaminergic medication is working and symptoms are well controlled). Demographic, general clinical and family data (sex, education, age, handedness, age at onset, PD duration, and family history) were obtained using a semi-structured interview. Levodopa equivalent daily dose (LEDD)^48^ was calculated. Disease severity was defined using the HY stage score^49^ and the UPDRS-III^50^. The presence and the severity of FoG was defined according to both the observation of FoG by a neurologist (item 3.11 UPDRS-III ≥ 1) and the FoG Questionnaire (item 3 of FoG-Q ≥ 2)^51^. Motor experiences of daily living were assessed using UPDRS-II^50^. Non-motor complications such as dyskinesia and fluctuations were evaluated using UPDRS-IV^50^. Non-Motor Symptoms Questionnaire (NMS-Q) was used to assess non-motor symptoms (i.e., gastrointestinal, urinary, olfactory, orthostatic and sexual dysfunctions)^52^. The most prominent clinical features of REM Sleep Behavior Disorder were assessed with the RBD Screening Questionnaire (RBDSQ)^53^.

### Neuropsychological and behavioral evaluations

At study entry and each follow-up visit, patients performed neuropsychological and behavioral evaluations within 48 h from MRI by expert neuropsychologists, blinded to the neurological data and MRI results. All the neuropsychological and behavioral variables were acquired at each time point. The same test battery was applied in healthy controls at study entry. The assessment evaluated global cognition with the Addenbrooke’s Cognitive Examination-revised (ACE-R); memory with the Rey Auditory Verbal Learning Test (RAVLT), and the pattern (PRM) and spatial (SRM) recognition memory tests from the Cambridge Neuropsychological Test Automated Battery (CANTAB); executive functions with the digit span backward, Intra/Extra Dimensional Set Shift test (IED) from the CANTAB, and the Stroop color-word test; attention and working memory with the digit ordering test and the letter cancellation test; language with the Boston Naming Test (BNT) and the language subtest of ACE-R; language fluency with semantic and phonemic fluencies; visuospatial abilities with the Hooper Visual Organization test and the visuospatial subtest of ACE-R. Mood was evaluated with the Hamilton Depression Rating Scale score (HDRS), Hamilton Anxiety Rating scale score (HAMA) and Apathy Evaluation Scale. For neuropsychological references, see our previous works^11^.

### MRI acquisition

Baseline and follow-up brain MRI scans were acquired on the same 1.5 Tesla Philips Medical System Achieva machine. Subjects were scanned between 10 and 11 a.m., i.e., treated PD patients were 90–120 min after their regular morning dopaminergic therapy administration in order to be sure they were in a stable ON phase. The following MR sequences were obtained in the following order: 1) gradient-echo (GRE) echo planar imaging (EPI) for RS fMRI (TR = 3000 ms, TE = 35 ms, flip angle = 90°, matrix size=128 × 128, FOV = 240 × 240 mm^2^; slice thickness=4 mm, 200 sets of 30 contiguous axial slices). During RS fMRI scanning, subjects were instructed to remain motionless, to keep their eyes closed, and not to think about anything in particular; 2) high resolution 3D sagittal T1-weighted Turbo Field Echo (TFE) (frequency direction=anterior-posterior, TR = 7.1 ms, TE = 3.3 ms, inversion time = 1000 ms, flip angle = 8°, matrix size = 256 × 256 × 180 [inferior-superior, anterior-posterior], FOV = 256×256 mm^2^, section thickness = 1 mm; voxel size= 1 × 1× 1 mm^3^, out-of-plane SENSE parallel reduction factor = 1.5, sagittal orientation); and 3) dual-echo (DE) turbo spin-echo (repetition time [TR] = 3125 ms, echo time [TEs]=20/100 ms, echo train length = 6, 44 axial slices, thickness = 3.0 mm, matrix size = 256 × 247, field of view [FOV] = 240 × 232 mm^2^; voxel size, 0.94 × 0.94 ×3 mm^3^, in-plane sensitivity encoding [SENSE] parallel reduction factor, 1.5).

### MRI analysis

MRI analysis was performed at the Neuroimaging Research Unit, IRCCS San Raffaele Scientific Institute, Milan, Italy, by experienced observers, blinded to subjects’ identity. The presence of vascular abnormalities, including WM hyperintensities and lacunes, was checked on DE images.

#### Cortical thickness measurement

Cortical reconstruction and estimation of mean cortical thickness (34 regions of interest [ROIs] per hemisphere) based on gyral and sulcal structures^54^ were performed on the 3D T1-weighted TFE images using the FreeSurfer image analysis suite, version 5.3 (http://surfer.nmr.mgh.harvard.edu/)^55^ as previously described^11^ and the mean cortical thickness measures of each ROI were calculated.

#### Basal ganglia measurement

FMRIB’s Integrated Registration and Segmentation Tool (FIRST) in FSL (https://fsl.fmrib.ox.ac.uk/fsl/fslwiki/FIRST) was applied to 3D T1-weighted TFE images of each subject at each visit and used to automatically segment basal ganglia bilaterally. Mean basal ganglia volumes were calculated and multiplied by the normalization factor derived from SIENAx to correct for subject head size (http://www.fmrib.ox.ac.uk/fsl/sienax/index.html).

#### Graph metrics of functional brain networks

Global and mean lobar functional network characteristics were explored using the Brain Connectivity Matlab toolbox (http://www.brain-connectivity-toolbox.net). Eighty-three ROIs from both hemispheres were grouped into six anatomical macro-areas: temporal, parietal, occipital, fronto-insular, basal ganglia, and sensorimotor areas^56,57^. Network metrics (nodal strength, characteristic path length, local efficiency, clustering coefficient) were assessed to characterize the topological organization of global brain and lobar networks^20^. The strength of a node is defined as the sum of weights of links connected to the node. Path length is the average shortest path to reach another node. Clustering coefficient is how well a node tends to cluster and so to be segregated in the network or in the brain.

Local efficiency considers how well the node’s neighbors are linked to each other, so how well a node is integrated.

##### Brain parcellation

T1-weighted images were processed and parcellated using the Freesurfer suite (V 5.3 http://surfer.nmr.mgh.harvard.edu/), resulting in 83 areas, which were used to define the brain nodes for the network analysis. Briefly, the images were automatically processed following the standard freesurfer procedure, which includes brain extraction, segmentation of grey matter (GM) and white matter (WM), and parcellation in cortical and subcortical regions. GM was parcellated according to a standardized atlas^54^ and segmented in 68 cortical areas. Segmentations of basal ganglia were obtained from the Freesurfer pipeline and added to the previous 68 areas.

##### RS fMRI preprocessing

RS fMRI data processing was carried out using the FMRIB software library (FSLv5.0). First, T1-weighted images were skull stripped using the Brain Extraction Tool and segmented in GM, WM, and cerebrospinal fluid (CSF) maps using the FMRIB’s Automated Segmentation Tool. Resulting images were registered into the RS fMRI native space of each subject through a 7 degree-of freedom (DOF) linear affine transformation using FMRIB’s Linear Image Registration Tool. The first four volumes of the fMRI data were removed to reach complete magnet signal stabilization. Then, individual RS fMRI images were processed using MELODIC (Multivariate Exploratory Linear Optimized Decomposition into Independent Components; version 3.10; http://www.fmrib.ox.ac.uk/fsl/melodic/)^58^. The following FSL-standard preprocessing pipeline was applied: (1) motion correction using MCFLIRT; (2) high-pass temporal filtering (lower frequency: 0.01 Hz); (3) spatial smoothing (Gaussian Kernel of FWHM 6 mm). Head motion parameters (mean absolute cumulative translation and mean rotation) are reported in Supplementary Table 1. Differences between PD patients and healthy controls and between PD groups at baseline were assessed using Kruskal–Wallis test. Linear regression models were built to assess longitudinal head motion parameter changes. Head motion parameters were considered as the dependent variable in each model, which also included individual follow-up duration as covariate (independent variable). Baseline analyses reported no significant differences in the amount of head movement in PD subgroups and controls (Supplementary Table 1). PD groups did not show any significant change in the amount of head movement over time (beta values ranging from 0 to 0.13; p values ranging from 0.33 to 1.00).

##### Functional connectome reconstruction

Functional connectivity matrices were obtained on the basis of correlation analysis. Mean time series were extracted from each region of interest by averaging the signal from all voxels within each region. RS fMRI data were masked with subject’s GM map in order to consider only voxels corresponding to GM and avoid the effect of atrophy. Cortical GM was segmented using SPM12, while basal ganglia (bilateral caudate, globus pallidus, putamen, and thalamus) maps were obtained using FIRST in FSL. The Pearson’s correlation coefficient between the mean time-series of each node pair, indicating the level of functional connectivity between regions *i* and *j*, was enter into cell *c*(*i*,*j*) of the matrix. Pearson’s correlation coefficients were then converted to *z*-scores using Fisher’s r-to-z transformation. Negative values were set as ‘NaN’ to mark these brain regions as unconnected^58^. Functional connections of each subject were required to be present in each structural connectivity matrix, i.e., we measured functional interactions only where an anatomical connection between two areas occurs in the independent healthy control sample. Indeed, functional connectome matrices are dense and we need to apply a threshold in order to avoid spurious functional connections. Since neuron-to-neuron transmission along network connections is the most likely mechanism for the pattern of pathological spread in neurodegenerative diseases, we decided to mask functional matrix with structural data. Consequently, we measured functional interactions only where anatomical connection is present. The construction of the white matter network in an independent healthy control group (*N* = 99, mean age = 51.22 ± 13.90, sex = 61 female and 38 male, mean MMSE = 29.72 ± 0.63) has been already described in our previous paper^20^.

### Statistical analysis

Socio-demographic and clinical variables, the mean cortical thickness of 34 ROIs per hemisphere, the mean basal ganglia volumes and graph metrics were compared between groups at the study entry using the Kruskal-Wallis test, followed by Dunn’s post-hoc test for continuous data, or Fischer’s exact test for categorical data. Linear mixed effect models were estimated in PD groups and group-by-time interaction was assessed to evaluate longitudinal between-group differences using time as a continuous variable. Random effect of subject for each model has been considered. Such models were adjusted for age, sex, LEDD at baseline and LEDD change over time.

Baseline MRI metrics which were found to be significantly different between groups were correlated with clinical data showing statistically significant changes over 1- and 2-years using Spearman’s correlation. Moreover, a ROC curve was calculated to identify the accuracy of clinical and MRI metrics in distinguishing PD-FoG-converters from PD-non-converters. Stepwise model selection procedure was applied to candidate baseline clinical and MRI metrics chosen a priori on the basis of variables that were significantly different in the following comparisons: (i) controls *vs* PD-FoG-converters; (ii) PD-non-converters *vs* PD-FoG-converters; and (ii) PD-non-converters *vs* PD-FoG. The approach used for the stepwise model was a bidirectional elimination, a combination of forward selection and backward elimination. We tested the multicollinearity between different predictors.

All statistical analyses were performed using SPSS (version 26) and R Statistical Software (version 4.0.3); *p* values were Bonferroni-corrected for the number of group comparisons at *p* < 0.05.

### Reporting summary

Further information on research design is available in the Nature Research Reporting Summary linked to this article.

## Supplementary information

Supplementary material

Reporting Summary

## Data Availability

The dataset used and analyzed during the current study will be made available by the corresponding author upon request to qualified researchers (i.e., affiliated to a university or research institution/hospital).
