# Supplementary material for: MRI biomarkers of freezing of gait development in Parkinson’s disease
Source: NPJ Parkinsons Dis. 2022 Nov 15;8:158. doi: 10.1038/s41531-022-00426-4 (PMC9666554; doi:10.1038/s41531-022-00426-4)
Supplement: Supplementary file 1 — Supplementary material [file 41531_2022_426_MOESM1_ESM.docx]

**SUPPLEMENTARY MATERIAL**

**Supplementary Table 1.** Head motion parameters at study entry in PD groups and HC.

| **Variables** | **HC** | **PD-non-converters** | **PD-FoG-converters** | **PD-FoG** | **P overall** | **p:**  **PD-non converters vs HC** | **p:**  **PD-FoG-converters vs HC** | **p:**  **PD-FoG vs HC** | **p:**  **PD-non converters vs PD-FoG-converters** | **p:**  **PD-non converters vs PD-FoG** | **p:**  **PD-FoG-converters vs PD-FoG** |
| --- | --- | --- | --- | --- | --- | --- | --- | --- | --- | --- | --- |
| **Mean absolute cumulative translation**  **[mm]** | -0.001 ± 0.211 | 0.000 ± 0.004 | 0.004 ± 0.093 | 0.121 ± 0.488 | 0.16 | 1.00 | 0.71 | 1.00 | 1.00 | 1.00 | 0.15 |
| **Mean rotation**  **[°]** | 0.001 ± 0.006 | -0.015 ± 0.179 | 0.001 ± 0.004 | -0.001 ± 0.006 | 0.60 | 1.00 | 1.00 | 1.00 | 1.00 | 1.00 | 1.00 |

Values are reported as mean ± standard deviation. Differences between PD groups and healthy controls and between PD groups at baseline were assessed using Kruskal-Wallis test. Abbreviations: FoG= freezing of gait; HC= healthy controls; mm= millimeters; N= number; PD= Parkinson’s disease.
