# Supplementary material for: MRI biomarkers of freezing of gait development in Parkinson’s disease
Source: NPJ Parkinsons Dis. 2022 Nov 15;8:158. doi: 10.1038/s41531-022-00426-4 (PMC9666554; doi:10.1038/s41531-022-00426-4)
Supplement: Supplementary file 2 — Reporting Summary [file 41531_2022_426_MOESM2_ESM.pdf]

nature portfolio

Corresponding author(s): Federica Agosta

Last updated by author(s): Feb 28, 2022

# Reporting Summary

Nature Portfolio wishes to improve the reproducibility of the work that we publish. This form provides structure for consistency and transparency in reporting. For further information on Nature Portfolio policies, see our Editorial Policies and the Editorial Policy Checklist.

## Statistics

For all statistical analyses, confirm that the following items are present in the figure legend, table legend, main text, or Methods section.

| n/a | Confirmed | |
|---|---|---|
| ☐ | ☒ | The exact sample size (*n*) for each experimental group/condition, given as a discrete number and unit of measurement |
| ☒ | ☐ | A statement on whether measurements were taken from distinct samples or whether the same sample was measured repeatedly |
| ☐ | ☒ | The statistical test(s) used AND whether they are one- or two-sided<br>*Only common tests should be described solely by name; describe more complex techniques in the Methods section.* |
| ☐ | ☒ | A description of all covariates tested |
| ☐ | ☒ | A description of any assumptions or corrections, such as tests of normality and adjustment for multiple comparisons |
| ☐ | ☒ | A full description of the statistical parameters including central tendency (e.g. means) or other basic estimates (e.g. regression coefficient) AND variation (e.g. standard deviation) or associated estimates of uncertainty (e.g. confidence intervals) |
| ☐ | ☒ | For null hypothesis testing, the test statistic (e.g. *F*, *t*, *r*) with confidence intervals, effect sizes, degrees of freedom and *P* value noted<br>*Give P values as exact values whenever suitable.* |
| ☒ | ☐ | For Bayesian analysis, information on the choice of priors and Markov chain Monte Carlo settings |
| ☒ | ☐ | For hierarchical and complex designs, identification of the appropriate level for tests and full reporting of outcomes |
| ☒ | ☐ | Estimates of effect sizes (e.g. Cohen's *d*, Pearson's *r*), indicating how they were calculated |

*Our web collection on statistics for biologists contains articles on many of the points above.*

## Software and code

Policy information about availability of computer code

| | |
|---|---|
| Data collection | no sotware was used |
| Data analysis | FMRIB's Integrated Registration and Segmentation Tool (FIRST) in FSL, FreeSurfer image analysis suite, version 5.3, Brain Connectivity Matlab toolbox, SPSS (version 26) and R Statistical Software (version 4.0.3) |

For manuscripts utilizing custom algorithms or software that are central to the research but not yet described in published literature, software must be made available to editors and reviewers. We strongly encourage code deposition in a community repository (e.g. GitHub). See the Nature Portfolio guidelines for submitting code & software for further information.

## Data

Policy information about availability of data

All manuscripts must include a data availability statement. This statement should provide the following information, where applicable:

- Accession codes, unique identifiers, or web links for publicly available datasets
- A description of any restrictions on data availability
- For clinical datasets or third party data, please ensure that the statement adheres to our policy

The dataset used and analyzed during the current study will be made available by the corresponding author upon request to qualified researchers (i.e., affiliated to a university or research institution/hospital).

# Field-specific reporting

Please select the one below that is the best fit for your research. If you are not sure, read the appropriate sections before making your selection.

☒ Life sciences ☐ Behavioural & social sciences ☐ Ecological, evolutionary & environmental sciences

For a reference copy of the document with all sections, see nature.com/documents/nr-reporting-summary-flat.pdf

# Life sciences study design

All studies must disclose on these points even when the disclosure is negative.

| | |
|---|---|
| Sample size | N.A. |
| Data exclusions | Hoehn and Yahr (HY) score > 4 and/or dementia, moderate/severe head tremor at rest; cerebrovascular disorders (including vascular parkinsonism) or intracranial masses on routine MRI; history of traumatic brain injury; any other major neurological and medical condition; and MR images with artefacts. |
| Replication | no replication |
| Randomization | N.A. |
| Blinding | At study entry and at each follow-up visit, an experienced neurologist blinded to MRI results performed clinical assessments. Moreover, expert neuropsychologists, blinded to clinical and MRI results, performed neuropsychological and behavioral evaluations at each visit in both PD patients and healthy controls. Finally, MRI analysis was performed by two experienced observers, blinded to subjects' identity and diagnosis. |

# Reporting for specific materials, systems and methods

We require information from authors about some types of materials, experimental systems and methods used in many studies. Here, indicate whether each material, system or method listed is relevant to your study. If you are not sure if a list item applies to your research, read the appropriate section before selecting a response.

## Materials & experimental systems

| n/a | Involved in the study | |
|---|---|---|
| ☒ | ☐ | Antibodies |
| ☒ | ☐ | Eukaryotic cell lines |
| ☒ | ☐ | Palaeontology and archaeology |
| ☒ | ☐ | Animals and other organisms |
| ☐ | ☒ | Human research participants |
| ☒ | ☐ | Clinical data |
| ☒ | ☐ | Dual use research of concern |

## Methods

| n/a | Involved in the study | |
|---|---|---|
| ☒ | ☐ | ChIP-seq |
| ☒ | ☐ | Flow cytometry |
| ☐ | ☒ | MRI-based neuroimaging |

## Human research participants

Policy information about studies involving human research participants

| | |
|---|---|
| Population characteristics | The sample included 30 PD-FoG patients, 11 PD-FoG-converters and 11 PD-non-converters, matched for age, sex, education, disease duration and disease severity using the Unified Parkinson's Disease Rating Scale part III (UPDRS-III). PD-FoG patients had FoG at baseline, while PD-FoG-converters developed FoG over the 2-year follow-up. Patients received a comprehensive evaluation in ON medication state including neurological, cognitive/behavioral and MRI assessments at study entry and at 1-year and 2-year follow-up. |
| Recruitment | PD patients were prospectively recruited at the Clinic of Neurology, School of Medicine, University of Belgrade, Belgrade, Serbia within the framework of an ongoing longitudinal project |
| Ethics oversight | The study received approval from the ethics committee on human experimentation of Faculty of Medicine - University of Belgrade (No. 175090). Written informed consent was obtained from all patients participating in the study. |

Note that full information on the approval of the study protocol must also be provided in the manuscript.

# Magnetic resonance imaging

## Experimental design

| | |
|---|---|
| Design type | Longitudinal |
| Design specifications | Patients were assessed by brain MRI evaluations at study entry (baseline) and every year for two years. |
| Behavioral performance measures | N.A. |

## Acquisition

| | |
|---|---|
| Imaging type(s) | functional and structural |
| Field strength | 1.5 T |
| Sequence & imaging parameters | dual-echo (DE) turbo spin-echo, high resolution 3D sagittal T1-weighted Turbo Field Echo (TFE), gradient-echo (GRE) echo planar imaging (EPI) for RS fMRI |
| Area of acquisition | Whole brain |

Diffusion MRI ☐ Used ☒ Not used

## Preprocessing

| | |
|---|---|
| Preprocessing software | FSL and SPM |
| Normalization | NA |
| Normalization template | T1-weighted images were processed and parcellated using the Freesurfer suite (V 5.3 http://surfer.nmr.mgh.harvard.edu/), resulting in 83 areas, which were used to define the brain nodes for the network analysis. |
| Noise and artifact removal | Individual RS fMRI images were processed using MELODIC (Multivariate Exploratory Linear Optimized Decomposition into Independent Components; version 3.10; http://www.fmrib.ox.ac.uk/fsl/melodic/).2 The following FSL-standard preprocessing pipeline was applied: (1) motion correction using MCFLIRT; (2) high-pass temporal filtering (lower frequency: 0.01 Hz); (3) spatial smoothing (Gaussian Kernel of FWHM 6 mm); (4) single-session independent component analysis (ICA). |
| Volume censoring | The first four volumes of the fMRI data were removed to reach complete magnet signal stabilization. |

## Statistical modeling & inference

| | |
|---|---|
| Model type and settings | Linear mixed effect models were estimated in PD groups and group-by-time interaction was assessed to evaluate longitudinal between-group differences using time as a continuous variable. Baseline MRI metrics which were found to be significantly different between groups were correlated with clinical data changes over 1- and 2-years in PD-FoG and PD-FoG-converters using Spearman's correlation. Moreover, a ROC curve was calculated to identify the accuracy of clinical and MRI metrics in distinguishing PD-FoG-converters from PD-non-converters. |
| Effect(s) tested | NA |

Specify type of analysis: ☐ Whole brain ☐ ROI-based ☒ Both

| | |
|---|---|
| Anatomical location(s) | *Describe how anatomical locations were determined (e.g. specify whether automated labeling algorithms or probabilistic atlases were used).* |
| Statistic type for inference (See Eklund et al. 2016) | NA |
| Correction | p values were Bonferroni-corrected for multiple comparison at p<0.05 |

## Models & analysis

| n/a | Involved in the study |
|---|---|
| ☐ | ☒ Functional and/or effective connectivity |
| ☐ | ☒ Graph analysis |
| ☐ | ☒ Multivariate modeling or predictive analysis |

| | |
|---|---|
| Functional and/or effective connectivity | Functional connectivity matrices were obtained on the basis of correlation analysis. Mean time series were extracted from each region of interest by averaging the signal from all voxels within each region. |
| Graph analysis | Network metrics (nodal strength, characteristic path length, local efficiency, clustering coefficient) were assessed to characterize the topological organization of global brain and lobar networks. |
| Multivariate modeling and predictive analysis | a ROC curve was calculated to identify the accuracy of clinical and MRI metrics in distinguishing PD-FoG-converters from PD-non-converters |
